# Profiling Levels of Serum microRNAs and Soluble ACE2 in COVID-19 Patients

**DOI:** 10.3390/life12040575

**Published:** 2022-04-12

**Authors:** Noha Mousaad Elemam, Hind Hasswan, Hayat Aljaibeji, Narjes Saheb Sharif-Askari, Rabih Halwani, Jalal Taneera, Nabil Sulaiman

**Affiliations:** 1 Sharjah Institute for Medical Research, College of Medicine, University of Sharjah, Sharjah 27272, United Arab Emirates; nelemam@sharjah.ac.ae (N.M.E.); u00029330@sharjah.ac.ae (H.H.); nsharifaskari@sharjah.ac.ae (N.S.S.-A.); rhalwani@sharjah.ac.ae (R.H.); jtaneera@sharjah.ac.ae (J.T.); 2 Division of Endocrinology, Diabetes and Hypertension, Department of Medicine, Brigham and Women’s Hospital, Harvard Medical School, Boston, MA 02115, USA; haljaibeji@bwh.harvard.edu; 3 Department of Clinical Sciences, College of Medicine, University of Sharjah, Sharjah 27272, United Arab Emirates; 4 Prince Abdullah Ben Khaled Celiac Disease Chair, Department of Pediatrics, Faculty of Medicine, King Saud University, Riyadh 11451, Saudi Arabia; 5 Department of Basic Medical Sciences, College of Medicine, University of Sharjah, Sharjah 27272, United Arab Emirates; 6 Department of Family Medicine, College of Medicine, University of Sharjah, Sharjah 27272, United Arab Emirates; 7 Baker/IDI Heart and Diabetes Institute, Melbourne 3004, Australia

**Keywords:** miRNAs, sACE2, COVID-19, obesity, diabetes

## Abstract

**Background:** The main mechanism of viral entry in COVID-19 infection is through the angiotensin-converting enzyme 2 (ACE2) receptor present in the lungs. Numerous studies suggested a clinical significance of risk factors, such as gender, obesity, and diabetes on the soluble form of ACE2 (sACE2) and related miRNAs in COVID-19 infection. This study aims to investigate the serum level of sACE2 and 4 miRNAs (miR-421, miR-3909, miR-212-5p, and miR-4677-3p) in COVID-19 patients and assess their associations with clinicopathological parameters. **Methods:** Serum samples were collected from non-diabetic and diabetic COVID-19 patients and healthy controls. sACE2 levels were quantified using ELISA, and serum miRNA levels were measured using qPCR. In addition, laboratory blood tests were retrieved from the clinical records of COVID-19 patients. **Results:** sACE2 levels were upregulated in COVID-19 patients regardless of sex, diabetes status, or obesity. Furthermore, the four investigated miRNAs were upregulated in COVID-19 patients and were positively correlated with each other. Furthermore, miR-421, miR-3909, and miR-4677-3p were positively associated with sACE2, suggesting a strong link between these markers. Notably, miR-212-5p was selectively upregulated in moderate, male, and non-obese COVID-19 patients. Interestingly, miR-212-5p was correlated with D-dimer, while sACE2 was correlated with coagulation tests, such as aPTT and platelets, indicating their potential as markers of coagulopathy in COVID-19. Additionally, there was a positive correlation between sACE2 and C-reactive protein in diabetic COVID-19 patients, indicating a promising role of this marker in the inflammatory status of these patients. **Conclusion:** sACE2 and its regulatory miRNAs were upregulated and correlated with laboratory investigations of COVID-19 patients, thus indicating their clinical significance as biomarkers in COVID-19 infection.

## 1. Introduction

Despite the advanced research studies about the coronavirus disease 2019 (COVID-19), a gap still exists in understanding the various factors and comorbidities affecting this disease. COVID-19, caused by severe acute respiratory syndrome coronavirus-2 (SARS-CoV-2), was found to be affected by multiple factors and comorbidities, including gender, obesity, and type 2 diabetes (T2D) [1,2]. One of the mechanisms of SARS-CoV-2 viral entry is the host receptor angiotensin-converting enzyme 2 (ACE2) [3,4]. ACE2 receptor is highly expressed on epithelial cells of the lung, kidney, and vascular endothelium [5]. Due to this pandemic disease, several studies have shed light on the expression of ACE2 in the lung and pancreas tissues of diabetic patients, which was reported to increase their susceptibility to COVID-19 infection [6,7]. It is worth mentioning that ACE2 receptor can be cleaved into a soluble form (sACE2) using metalloproteinases [8]. Previously, we have reported a reduction in the circulating serum levels of sACE2 in non-infected patients with T2D [9]. In COVID-19, sACE2 showed controversial patterns, where some studies reported its levels to be higher or lower in the sera of patients, with variable levels according to disease severities [10,11]. However, sACE2 remains a crucial contributor to COVID-19 clinical outcomes and severity [12], which can be a promising therapeutic target in COVID-19 infection [13,14,15].

MicroRNAs (miRNAs) are non-coding, short, single-stranded RNA molecules that regulate gene expression through post-transcriptional and translation alteration [16]. Numerous miRNAs were implicated in COVID-19 prognosis, response to therapy, and mortality as well as being potential therapeutic targets [17,18].

Several research studies highlight that ACE2 expression is regulated by several miRNAs in various tissues. miRNAs such as miR-5197-3p, miR-17-5p. and miR-20b-5p were shown to directly affect the SARS-CoV-2 genome and inhibit its post-transcriptional expression [19,20]. In addition, other miRNAs such as miR-421 were reported to regulate human coronaviruses through direct binding to the viral genome [20].

Noteworthy, several serum miRNAs showed great potential as biomarker candidates in various diseases. Formerly, four miRNAs (miR-421, miR-3909, miR-212-5p, and miR-4677-3p) were identified via an in-silico approach using four different tools as potential upstream regulators of ACE2 by targeting the 3′-untranslated region (UTR) [9]. Therefore, this study aimed to investigate the serum levels of sACE2 and these four upstream miRNAs in COVID-19 patients with a particular interest in diabetes as a comorbidity.

## 2. Subjects, Materials, and Methods

### 2.1. Subjects

A total of 119 subjects were recruited in this study, including 60 healthy controls and 59 COVID-19 patients. Included COVID-19 patients visited Rashid Hospital, Dubai, UAE, from May–July 2020, and the laboratory confirmed SARS-CoV-2 infection by reverse transcription-quantitative polymerase chain reaction (RT-qPCR). The patients’ characteristics were extracted from the study subjects’ clinical records. Out of the 59 COVID-19 patients, 27 patients had diabetes as comorbidity, while 32 patients were not diabetic. The collected data included age, gender, BMI, COVID-19 severity and symptoms (fever, headache, flu, dyspnea, myalgia, diarrhea, cough, vomiting, nausea, loss of smell, loss of taste, body ache, and sore throat), and comorbidities (such as hypertension, dyslipidemia, asthma, allergy, renal insufficiency, and cardiovascular diseases). The diabetic status of COVID-19 patients was determined based on physicians’ previous diagnosis. This was done using measurements of fasting blood glucose (FBG), HbA1c, and participants’ clinical records. Following the WHO criteria for both of FBG and HbA1c, normal: FBG  <  6.1 mmol/l, impaired fasting glucose (IFG): 6.1–6.9 mmol/l, and diabetes: ≥ 7.0 mmol/l, whereas HbA1c < 6.5% was considered non-diabetic and ≥ 6.5% was diagnostic of diabetes, as previously described [21].

In addition, laboratory tests were collected, including absolute neutrophil count (ANC), absolute lymphocyte count (ALC), ANC/ALC ratio, C-reactive protein (CRP), creatinine, alanine transaminase (ALT), aspartate aminotransferase (AST), D-Dimer, triglycerides, ferritin, prothrombin time (PT), activated partial thromboplastin time (aPTT), lactate dehydrogenase (LDH), blood urea nitrogen (BUN), albumin, bilirubin, gamma-glutamyl transpeptidase (GGT), hemoglobin (Hb), platelets, and white blood cell count (WBC). COVID-19 patients were classified into 3 categories: asymptomatic, mild, and moderate. The asymptomatic group included those patients with positive SARS-CoV-2 test and no reported symptoms. Mild cases were those patients with uncomplicated upper respiratory tract infection in addition to non-specific symptoms, such as sore throat, dry cough, fever, myalgia, arthralgia, and runny nose without shortness of breath. Moderate COVID-19 patients are those cases with pneumonia along with symptoms of fever, cough, dyspnea, and fast breathing [22]. Table 1 summarizes the demographic, laboratory, symptoms, and clinical data of COVID-19 patients. The ethical approval was obtained by the ethical committee at Rashid Hospital in Dubai (DSREC-08/2021-14). All participants signed an informed consent form before blood sample and data collection.

### 2.2. Serum Collection and RNA Extraction

Serum was obtained from whole-blood samples by centrifugation and was stored at −80 °C until it was used for RNA extraction and Enzyme-Linked Immunosorbent Assay (ELISA). RNA extraction was performed using 200 μls of serum and miRNeasy Serum/Plasma Kit (Qiagen, Hilden, Germany), as per manufacturer’s instructions. RNA was eluted in 20 μls of nuclease-free water.

### 2.3. Human Serum miRNA Quantification

Reverse transcription for each of the miRNAs was performed using High-Capacity cDNA Reverse Transcription Kit (ThermoFisher Scientific, Waltham, MA, USA) and TaqMan microRNA assay reagents (Applied Biosystems, Foster City, CA, USA) for each of the 4 miRNAs: miR-421, miR-3909, miR-212-5p, and miR-4677-3p.

miRNA expression levels were quantified using respective target primers: FAM tagged miR-421 (Assay ID: 002700), miR-3909 (Assay ID: 465180_mat), miR-212-5p (Assay ID: 461768_mat), and miR-4677-3p (Assay ID: 465015_mat), along with 5x HOT FIREPol Probe Universal qPCR Mix (SolisBioDyne, Tartu, Estonia). qRT-PCR was performed using QuantStudio 3 Real-time qPCR (Applied Biosystems, Foster City, CA, USA). The relative quantification (RQ) of miRNA expression levels was calculated using the equation (RQ = 2^−ΔCT^), where ΔCT is calculated based on the average CT values of the healthy controls, as previously described [9].

### 2.4. Quantification of sACE2 Using ELISA

The levels of sACE2 in the serum of healthy controls and COVID-19 patients were assessed using the Human ACE2 DuoSet ELISA (R&D Systems, Minneapolis, MN, USA), according to the manufacturer’s instructions.

### 2.5. Statistical Analysis

Statistical analyses were done using GraphPad Prism 6 (GraphPad Software, San Diego, CA, USA). All the variables are expressed as mean ± standard error of the mean (SEM). The data were subjected to normality tests, after which the comparisons were analyzed using the Mann–Whitney U-test, while the correlation analysis was performed using Spearman’s method. *p* < 0.05 was considered statistically significant.

## 3. Results

### 3.1. Serum sACE2 Levels Are Increased in COVID-19 Patients with Different Severities

**Figure 1:**
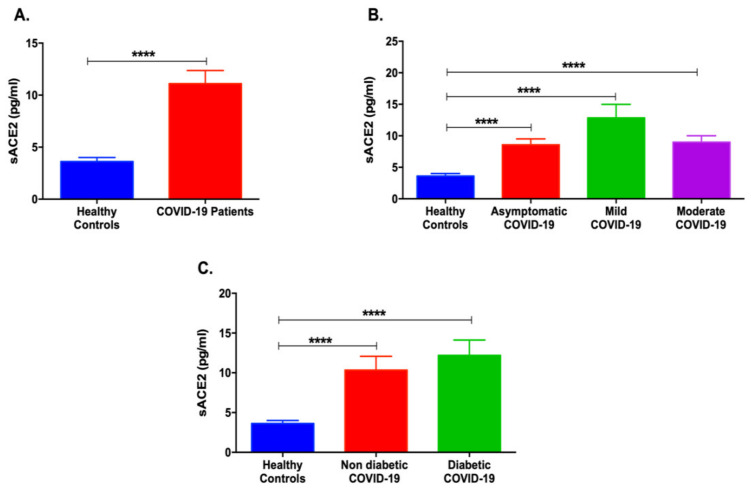
Serum levels of sACE2 in COVID-19 patients and healthy controls. (**A**) sACE2 was significantly elevated in COVID-19 patients compared to healthy controls. (**B**) Mild, moderate, and asymptomatic COVID-19 patients showed an increase in sACE2 compared to healthy controls. (**C**) Both non-diabetic and diabetic COVID-19 patients showed a rise in sACE2 levels. Data are represented as mean ± standard error of mean (SEM). **** *p* < 0.0001.

First, it was of interest to explore the serum levels of sACE2 in COVID-19 patients. As shown in Figure 1A, a significant increase in the levels of sACE2 was found in COVID-19 patients compared to healthy individuals (*p* < 0.0001). Moreover, upon the classification of COVID-19 patients according to their disease severity, sACE2 was elevated in mild, moderate, and asymptomatic COVID-19 patients (*p* < 0.0001, Figure 1B). On another note, non-diabetic and diabetic COVID-19 patients showed increased levels of sACE2 compared to healthy individuals (Figure 1C).

### 3.2. Serum Levels of miR-421 and miR-3909 Are Higher in COVID-19 Patients

**Figure 2:**
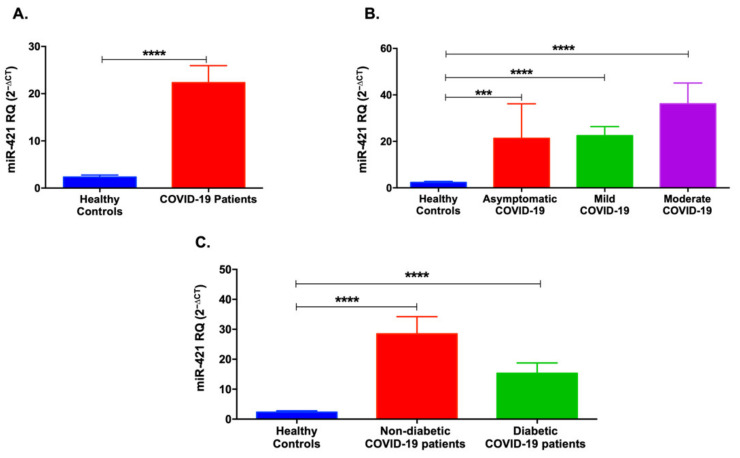
Serum levels of miR-421 in COVID-19 patients and healthy controls. (**A**) miR-421 was significantly upregulated in COVID-19 patients compared to healthy controls. (**B**) Mild, moderate, and asymptomatic COVID-19 patients showed an increase in miR-421 levels. (**C**) Non-diabetic and diabetic COVID-19 patients showed an upregulation of miR-421 levels. Data are represented as mean ± standard error of mean (SEM). *** *p* < 0.001 and **** *p* < 0.0001.

Similarly, miR-421 serum levels were elevated in recruited COVID-19 patients, as shown in Figure 2A (*p* < 0.0001). In particular, several stage severities, including asymptomatic, mild, and moderate COVID-19 patients, showed an upregulation in miR-421 levels compared to healthy individuals (*p* < 0.001, Figure 2B). In addition, as illustrated in Figure 2C, both diabetic and non-diabetic COVID-19 patients showed a significant increase in the serum miR-421 levels compared to healthy individuals, with a higher elevation in the non-diabetic COVID-19 patients (*p* < 0.0001).

**Figure 3:**
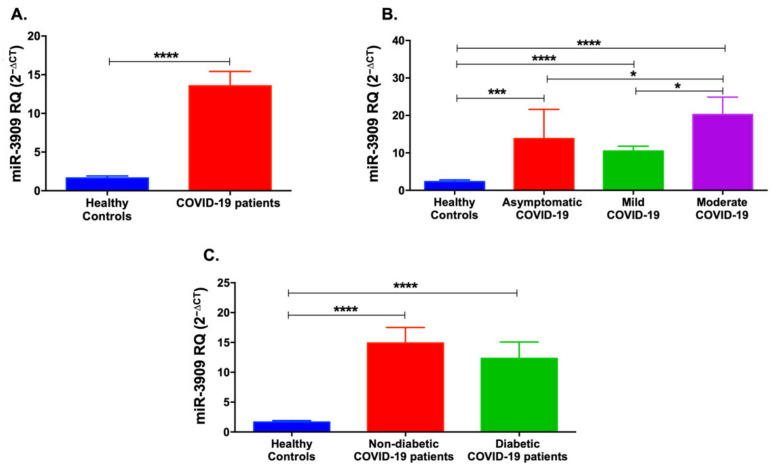
Serum levels of miR-3909 in COVID-19 patients and healthy controls. (**A**) miR-3909 was significantly upregulated in COVID-19 patients compared to healthy controls. (**B**) Mild, moderate, and asymptomatic COVID-19 patients showed an increase in miR-3909 levels. (**C**) Non-diabetic and diabetic COVID-19 patients showed an upregulation of miR-3909 levels. Data are represented as mean ± standard error of mean (SEM). * *p* < 0.05, *** *p* < 0.001, and **** *p* < 0.0001.

Likewise, miR-3909 levels were elevated in COVID-19 patients cumulatively (*p* < 0.0001, Figure 3A) as well as in the mild (*p* < 0.0001), moderate (*p* < 0.0001), and asymptomatic (*p* < 0.001) patients (Figure 3B). Further, miR-3909 was elevated in moderate COVID-19 patients compared to asymptomatic or mild COVID-19 cases (*p* < 0.05). Additionally, miR-3909 was upregulated in non-diabetic and diabetic COVID-19 patients (*p* < 0.0001, Figure 3C).

### 3.3. Serum Levels of miR-212-5p and miR-4677-3p Are Higher in COVID-19 Patients and Are Affected by Disease Severity

**Figure 4:**
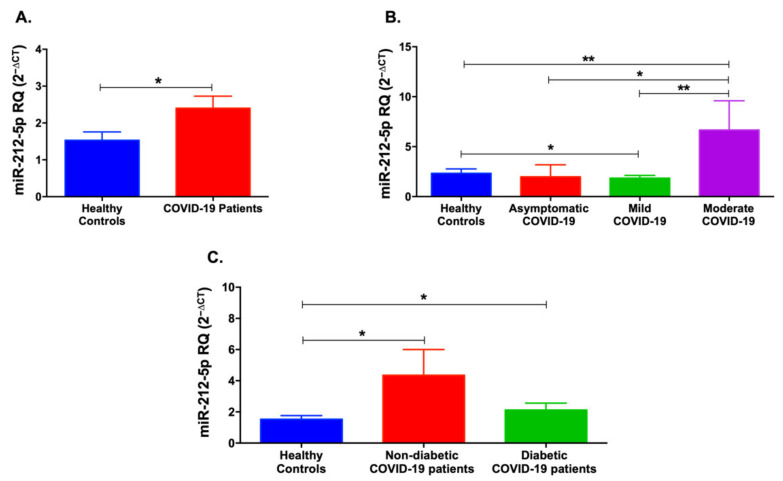
Serum levels of miR-212-5p levels in COVID-19 patients and healthy controls. (**A**) miR-212-5p was significantly upregulated in COVID-19 patients compared to healthy controls. (**B**) Moderate COVID-19 patients showed an increase in miR-212-5p levels compared to healthy controls and mild COVID-19 patients. (**C**) Non-diabetic and diabetic COVID-19 patients showed an upregulation of miR-212-5p levels. Data are represented as mean ± standard error of mean (SEM). * *p* < 0.05 and ** *p* < 0.01.

miR-212-5p was significantly upregulated in COVID-19 patients (*p* < 0.05, Figure 4A). However, stratification of the patients into mild, moderate, and asymptomatic revealed a significant elevation only in the moderate COVID-19 patients compared to healthy controls (*p* < 0.01, Figure 4B). Additionally, moderate COVID-19 patients showed higher levels of serum miR-212-5p when compared to asymptomatic and mild COVID-19 patients (*p* < 0.05 and *p* < 0.01, respectively). On the contrary, there was a decrease in miR-212-5p levels in serum of mild COVID-19 patients compared to healthy controls (*p* < 0.05). As shown in Figure 4C, when comparing the diabetic status of COVID-19 patients, both non-diabetic and diabetic COVID-19 patients showed increased levels of miR-212-5p compared to healthy controls (*p* < 0.05).

**Figure 5:**
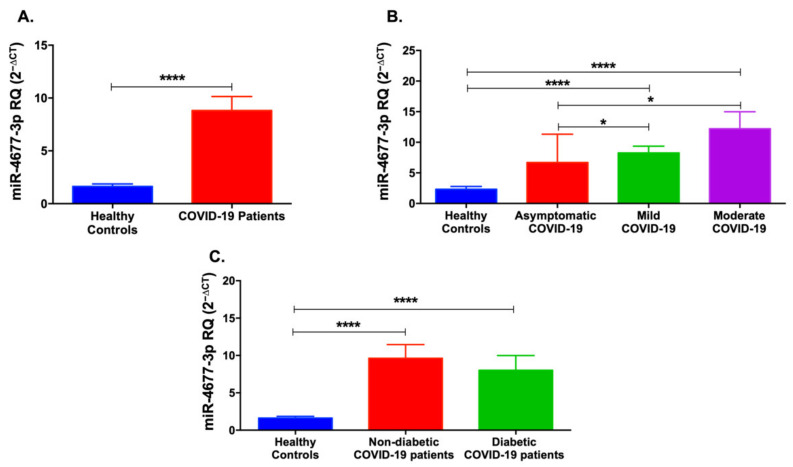
Serum levels of miR-4677-3p levels in COVID-19 patients and healthy controls. (**A**) miR-4677-3p was significantly upregulated in COVID-19 patients compared to healthy controls. (**B**) Mild and moderate COVID-19 patients showed an increase in miR-4677-3p levels. (**C**) Non-diabetic and diabetic COVID-19 patients showed an upregulation of miR-4677-3p levels. Data are represented as mean ± standard error of mean (SEM). * *p* < 0.05 and **** *p* < 0.0001.

miR-4677-3p was also significantly upregulated in COVID-19 patients (*p* < 0.0001, Figure 5A). Interestingly, miR-4677-3p levels were elevated in mild and moderate COVID-19 patients compared to healthy controls (*p* < 0.0001), with a significant increase in mild and moderate COVID-19 patients when compared to asymptomatic patients (*p* < 0.05, Figure 5B). Likewise, miR-4677-3p was upregulated in non-diabetic and diabetic COVID-19 patients (*p* < 0.0001, Figure 5C).

### 3.4. Impact of Gender Variation on the Expression of sACE2, miR-421, miR-212-5p, miR-3909, and miR-4677-3p

**Figure 6:**
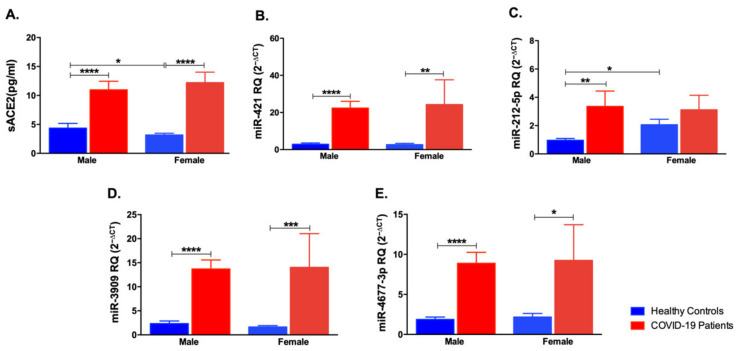
Impact of gender variation on sACE2, miR-421, miR-212-5p, miR-3909, and miR-4677-3p levels in COVID-19 patients and healthy controls. (**A**) sACE2, (**B**) miR-421, (**C**) miR-212-5p, (**D**) miR-3909, and (**E**) miR-4677-3p were compared in male and female COVID-19 patients and healthy controls. Data are represented as mean ± standard error of mean (SEM). * *p* < 0.05, ** *p* < 0.01, *** *p* < 0.001, and **** *p* < 0.0001.

As mentioned earlier, gender was a critical factor in COVID-19 pathogenesis. Hence, it was vital to compare the levels of sACE2 and investigate miRNAs in female and male individuals. As shown in Figure 6A, sACE2 was increased in both male and female COVID-19 patients compared to the controls (*p* < 0.0001). Further, healthy female controls showed lower levels of sACE2 compared to healthy male controls (*p* < 0.05). miR-421 was found to be upregulated in male and female COVID-19 patients compared to the respective healthy controls (*p* < 0.0001 and *p* < 0.01, Figure 6B). Another interesting finding was the high levels of miR-212-5p in male COVID-19 patients and not the female group. Besides this finding, healthy female individuals had higher miR-212-5p than healthy male controls (*p* < 0.05, Figure 6C). The other miRNAs, miR-3909 and miR-4677-3p, were significantly upregulated in male COVID-19 patients compared to healthy controls (*p* < 0.0001) as well as in the female COVID-19 patients compared to respective controls (*p* < 0.001 and *p* < 0.05, respectively).

### 3.5. Impact of Body Mass Index Variation on the Expression of sACE2, miR-421, miR-212-5p, miR-3909, and miR-4677-3p

**Figure 7:**
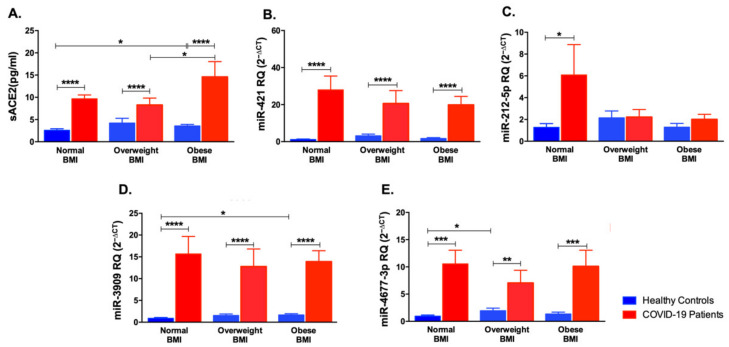
Impact of BMI variation on sACE2, miR-421, miR-212-5p, miR-3909 and miR-4677-3p levels in COVID-19 patients and healthy controls. (**A**) sACE2, (**B**) miR-421, (**C**) miR-212-5p, (**D**) miR-3909, and (**E**) miR-4677-3p were compared in normal, overweight, and obese COVID-19 patients and healthy controls. Data are represented as mean ± standard error of mean (SEM). * *p* < 0.05, ** *p* < 0.01, *** *p* < 0.001, and **** *p* < 0.0001.

Obesity was reported to affect the expression of ACE2 and its soluble form as well as these miRNAs [9]. Thus, the cohort was divided into three categories according to their body mass index (BMI), after which sACE2 and miRNAs expression was compared. As illustrated in Figure 7A, sACE2 was significantly increased in COVID-19 patients with normal, overweight, or obese BMI ranges (*p* < 0.0001) compared to healthy controls. Furthermore, obese healthy individuals were reported to have higher sACE2 than those with a normal BMI range (*p* < 0.05). In addition, obese COVID-19 patients had higher levels of sACE2 compared to overweight COVID-19 patients (*p* < 0.05). miR-421 was elevated in COVID-19 patients compared to healthy controls despite the variation in BMI (*p* < 0.0001). Surprisingly, miR-212-5p was found to be increased in COVID-19 patients with a normal BMI range compared to their respective healthy controls (*p* < 0.05). Moreover, miR-3909 was found to be elevated in all COVID-19 patients belonging to different BMI groups compared to healthy controls (*p* < 0.0001). Healthy controls with an obese BMI range had higher levels of miR-3909 compared to healthy individuals with a normal BMI range (*p* < 0.05). Finally, miR-4677-3p was found to be increased in COVID-19 patients with normal (*p* < 0.001), overweight (*p* < 0.01), and obese (*p* < 0.001) BMI ranges compared to the respective healthy controls. Furthermore, overweight healthy controls had higher levels of miR-4677-3p than healthy controls with normal BMI (*p* < 0.05). These results highlight the critical regulatory effect of BMI in COVID-19 patients.

### 3.6. Correlation Analysis between sACE2, miRNAs and the Clinical and Laboratory Investigations of COVID-19 Patients

It was critical to explore the association between the four investigated miRNAs: miR-421, miR-212-5p, miR-3909, and miR-4677-3p in the healthy controls and COVID-19 patients. As summarized in Table 2, positive correlations were observed between the level of the four miRNAs, which further supports the hypothesis of a possible link between the four miRNAs.

Furthermore, miR-421 (r = 0.581, *p* = 4.1x 10^−12^), miR-3909 (r = 0.670, *p* = 6.9 × 10^−17^), and miR-4677-3p (r = 0.448, *p* = 7.1 × 10^−7^) were positively correlated with sACE2, indicating a strong link between the serum levels of these miRNAs and sACE2. Interestingly, miR-421 (r = 0.371, *p* = 7 × 10^−5^), miR-3909 (r = 0.450, *p* = 8.6 × 10^−7^), miR-4677-3p (r = 0.340, *p* = 0.00046), and sACE2 (r = 0.480, *p* = 1.19 × 10^−7^) were positively correlated with the age of the recruited cohort. On the other hand, these three miRNAs and sACE2 were negatively correlated with the BMI of the recruited cohort, as summarized in Table 3.

To investigate the existence of any association between the laboratory tests of COVID-19 patients with the levels of the miRNAs and sACE2, Spearman’s correlation analysis was performed. As indicated in Table 4, miR-212-5p was found to be negatively associated with D-dimer tests in COVID-19 patients (r = −0.467, *p* = 0.0008) as well as in diabetic COVID-19 patients (r = -0.617, *p* = 0.0013). On the other hand, sACE2 was positively correlated with aPTT (r = 0.350, *p* = 0.0146) and platelets (r = 0.298, *p* = 0.033) in COVID-19 patients. Furthermore, sACE2 was positively correlated with platelets (r = 0.50, *p* = 0.01) in non-diabetic COVID-19 patients as well as CRP (r = 0.44, *p* = 0.024) and aPTT (r = 0.458, *p* = 0.021) in diabetic COVID-19 patients. Looking at diabetic COVID-19 patients, miR-421 was found to be positively correlated to ANC (r = 0.452, *p* = 0.02) and ANC/ALC ratio (r = 0.471, *p* = 0.015), while miR-3909 was found to show significant positive correlations with ANC (r = 0.573, *p* = 0.00219), ANC/ALC ratio (r = 0.513, *p* = 0.007), and WBC (r = 0.441, *p* = 0.024). Further, miR-4677-3p was found to be positively correlated with ANC/ALC ratio (r = 0.468, *p* = 0.018).

## 4. Discussion

The interaction between circulating sACE2 and regulating miRNAs in COVID-19 infection remains unclear. However, in this study, sACE2 and the four identified miRNAs: miR-421, miR-3909, miR-212-5p, and miR-4677-3p, were increased in COVID-19 patients. Furthermore, some of these markers were affected by disease severity, obesity, and gender. Moreover, sACE2 and miRNAs were associated with inflammatory and laboratory markers, highlighting their potential as biomarkers in COVID-19 infection.

sACE2 was found to be significantly upregulated in COVID-19 patients compared to healthy controls, which was in line with reports from different populations [11,12,23]. Furthermore, sACE2 was previously revealed to be higher in severe COVID-19 cases, especially those with respiratory distress, thus suggesting a host defense mechanism by competitive binding to the virus [23]. Additionally, such a mechanism was suggested by studies using recombinant ACE2 as a protective approach from COVID-19 infection [24,25]. In this study, sACE2 levels were not affected between female and male COVID-19 patients. Such findings contradict previous reports where males had higher levels of sACE2 than females. However, such populations were of a younger age group and male individuals with heart failure conditions, which could explain the different reported findings [10,20,26]. However, this study revealed higher sACE2 in healthy male individuals than females, supporting previous studies [27]. Further, sACE2 levels were increased in COVID-19 patients with or without T2D as comorbidity compared to healthy controls. As per our previous findings, we expected diabetic patients to have lower sACE2 levels than non-diabetics [9]; however, such a discrepancy could be attributed to the dominant effect of SARS-CoV-2 infection or the presence of other comorbidities, such as hypertension, or medications. The present study showed a significant increase in sACE2 in asymptomatic, mild, and moderate COVID-19 cases. However, there was no significant difference in the serum levels of sACE2 between the mild and moderate COVID-19 cases, which aligns with the previously reported data on mild and moderate COVID-19 patients that recovered from the infection [11]. It seems that sACE2 could further increase in severe COVID-19 patients, which is a limitation of the current study, as no severe/critical COVID-19 patients were included.

Previously, circulating sACE2 levels were reported to be higher in obesity, which could lead to comorbidities linked to COVID-19, and thus have an emerging role as a circulating biomarker for severity of outcome [28]. However, our current study showed that the sACE2 is upregulated in normal, overweight, and obese COVID-19 patients compared to healthy controls. Moreover, sACE2 was increased in obese compared to overweight COVID-19 patients, similar to our previous findings in healthy and diabetic individuals [9].

miRNAs, especially the circulating ones, have been described as crucial elements in COVID-19 infection [29,30]. However, little is still known about the four investigated miRNAs in this study. In addition to circulating sACE2, the explored upstream miRNAs (miR-421, miR-3909, miR-212-5p, and miR-4677-3p) were elevated in COVID-19 patients compared to healthy controls. The upregulation of serum miR-421 in COVID-19 aligns with previous studies where miR-421 was upregulated in inflammation [31]. On the other hand, the closely related miRNA, miR-421-5p, was indicated to be downregulated in hospitalized COVID-19 patients [32]. Interestingly, our results showed a tendency to decrease miR-421 levels in diabetic COVID-19 patients, which is in line with our previous reports [9].

miR-212-5p was upregulated in COVID-19 cases, particularly the moderate cases, and was unaffected by the diabetic status of the patients. Further, miR-212-5p levels were upregulated in moderate compared to mild cases of COVID-19 as well as being elevated in male but not female COVID-19 patients. Furthermore, miR-212-5p was found to be only upregulated in COVID-19 patients with normal BMI range but not in overweight or obese patients. Such findings indicate that miR-212-5p is strongly influenced by disease severity, gender, and obesity. In addition, miR-3909 was significantly upregulated in mild, moderate, and asymptomatic COVID-19 patients.

Similarly, miR-4677 levels were significantly upregulated in mild and moderate COVID-19 cases compared to healthy controls, with a differential expression between mild and asymptomatic COVID-19 patients. To our knowledge, the profile of miR-212-5p, miR-3909, and miR-4677-3p levels was not investigated before in COVID-19 infection. Moreover, these three miRNAs were unaffected by the diabetic status of COVID-19, similar to the previous reports in non-infected diabetic individuals [9].

The levels of the four investigated miRNAs in healthy and COVID-19 individuals were positively correlated with each other, suggesting a possible link between them. Further, the positive association between sACE2 and three of the explored miRNAs (miR-421, miR-3909, and miR-4677-3p) supports such a connection through the ACE2 receptor. This is the first study to investigate such correlation between sACE2 and these miRNAs. In addition, taking a closer look at COVID-19 patients, miR-212-5p was negatively correlated with D-dimer, thus highlighting a potential role in the thrombotic events observed in COVID-19 infection. Such a correlation is similar to the role of other miRNAs in thrombosis, such as miR-320a/b [33]. Likewise, sACE2 was correlated with coagulation tests, such as aPTT and platelets, but with a positive association, indicating its potential as a marker of coagulopathy in COVID-19. Additionally, the positive correlation between sACE2 and CRP in diabetic COVID-19 patients denoted a promising role of this marker in the inflammatory status of these patients.

## 5. Conclusions and Future Perspectives

This study pointed out the noticeable upregulation of the four miRNAs (miR-421, miR-212-5p, miR-3909, and miR-4677-3p) and the matching profiling of sACE2 in COVID-19 infection. In addition to their differential levels, this study explored the effect of factors such as disease severity, gender, and obesity on the levels of these markers in the serum as well as their association with laboratory tests, thus suggesting their potential as promising markers in COVID-19 infection. It is worth mentioning that this study has a few limitations, including limited sample size as well as the absence of severe/critical COVID-19 patients. Furthermore, ACE inhibitors (ACEIs) and angiotensin receptor blockers (ARBs) were previously reported to affect the expression of ACE2 as well as its soluble levels [34]. Such information was missing in the cohort included in this study. Future studies should further investigate the effect of such therapeutics on sACE2 and serum miRNA levels. Additionally, functional validation experiments would be essential to increase our understanding of the association between the four selected miRNAs and ACE2 in COVID-19 infection.

## Figures and Tables

**Table 1:** Demographic, clinical, and laboratory characteristics of COVID-19 patients. Continuous data are described as mean ± standard deviation (SD). ALC, absolute lymphocyte count; ALT, alanine transaminase; ANC, absolute neutrophil count; aPTT, activated partial thromboplastin time; AST, aspartate aminotransferase; BMI, body mass index; BUN, blood urea nitrogen; CRP, C-reactive protein, GGT, gamma-glutamyl transpeptidase; Hb, hemoglobin; LDH, lactate dehydrogenase; PT, prothrombin time; WBC, white blood cell count.

	COVID-19 Patients (*n* = 59)
**Disease Severity**	34 Mild 17 Moderate 8 Asymptomatic
**Gender (M/F)**	
Male	9/59
Female	50/59
**Age (Years)**	46.64 ± 14.20
**BMI (kg/m^2^)**	26.78 ± 4.865
**Comorbidities**	
Diabetes	27/59
Hypertension	19/59
Dyslipidemia	3/59
Cardiovascular Diseases	6/59
**Symptoms**	
Fever	37/59
Headache	3/56
Flu	1/59
Dyspnea	15/59
Myalgia	3/56
Diarrhea	5/59
Cough	28/59
Vomiting	6/59
Nausea	6/59
Loss Of Smell	0/59
Loss Of Taste	0/59
Body ache	3/59
Sore Throat	2/59
**Blood Tests**	
ANC (×103 cells/(L)	5.992 (3.64)
ALC (×103 cells/(L)	1.596 (0.820)
ANC/ALC ratio	5.698 (9.157)
Hb(g/dL)	12.9 (2.65)
WBC (×10^3^ cells/L)	8.52 (4.13)
**Liver Tests**	
ALT (U/L)	63.25 (108.7)
AST (U/L)	46.522 (67.68)
Albumin (g/dL)	4.37 (6.12)
Bilirubin (mg/dL)	0.982 (1.655)
GGT (U/L)	68.58 (54.47)
**Inflammatory Markers**	
CRP (mg/L)	65.3 (76.0)
D-Dimer (µg/mL)	1.35 (1.959)
Ferritin (ng/mL)	832.8 (948.4)
LDH (U/L)	312.3 (253.87)
**Renal Function Tests**	
Creatinine (mg/dL)	0.924 (0.427)
BUN (mg/dL)	27.53 (20.74)
Lipid Profile	
Triglycerides (mg/dL)	109.75 (51.03)
**Coagulation Tests**	
PT (seconds)	14.535 (1.577)
aPTT (seconds)	39.26 (6.65)
Platelets (×10^3^ cells/µL)	252.82 (122.98)

**Table 2:** Correlation analysis between the levels of miR-421, miR-212-5p, miR-3909, and miR-4677-3p in COVID-19 patients and healthy controls.

r; *p* Values	miR-421	miR-212-5p	miR-3909	miR-4677-3p
**miR-421**		0.484; 1.49 × 10^−7^	0.828; 3.73 × 10^−31^	0.824; 7.51 × 10^−29^
**miR-212-5p**	0.484; 1.49 × 10^−7^		0.391; 3.43 × 10^−5^	0.460; 1.00 × 10^−6^
**miR-3909**	0.828; 3.73 × 10^−31^	0.391; 3.43 × 10^−5^		0.708; 2.58 × 10^−18^
**miR-4677-3p**	0.824; 7.51 × 10^−29^	0.460; 1.00 × 10^−6^	0.708; 2.58 × 10^−18^	

**Table 3:** Correlation analysis between miR-421, miR-3909, miR-4677-5p, and sACE2 as well as age and BMI in COVID-19 patients and healthy controls.

	miR-421	miR-3909	miR-4677-3p	sACE2
**sACE2**	r = 0.581, *p* = 4.1x 10^−12^	r = 0.670, *p* = 6.9 × 10^−17^	r = 0.448, *p* = 7.1 × 10^−7^	-
**Age**	r = 0.371, *p* = 7 × 10^−5^	r = 0.450, *p* = 8.6 × 10^−7^	r = 0.340, *p* = 0.00046	r = 0.480, *p* = 1.19 × 10^−7^
**BMI**	r = −0.587, *p* = 1.1 × 10^−10^	r = −0.586, *p* = 1.2x 10^−10^	r = -0.459, *p* = 2.89 × 10^−6^	r = −0.599, *p* = 3.54 × 10^−11^

**Table 4:** Correlation analysis between sACE2, the four investigated miRNAs, and the laboratory tests of COVID-19 patients.

**COVID-19 Patients**	
miR-212-5p and D-Dimer	r = −0.467, *p* = 0.0008
sACE2 and aPTT	r = 0.350, *p* = 0.0146
sACE2 and Platelets	r = 0.298, *p* = 0.033
**Non-diabetic COVID-19 Patients**	
sACE2 and Platelets	r = 0.50, *p* = 0.01
**Diabetic COVID-19 Patients**	
miR-421 and ANC	r = 0.452, *p* = 0.02
miR-421 and ANC/ALC ratio	r = 0.471, *p* = 0.015
miR-212-5p and D-Dimer	r = −0.617, *p* = 0.0013
miR-3909 and ANC	r = 0.573, *p* = 0.00219
miR-3909 and WBC	r = 0.441, *p* = 0.024
miR-3909 and ANC/ALC ratio	r = 0.513, *p* = 0.007
sACE2 and CRP	r = 0.44, *p* = 0.024
sACE2 and aPTT	r = 0.458, *p* = 0.021
miR-4677-3p and ANC/ALC ratio	r = 0.468, *p* = 0.018

## Data Availability

Not Applicable.
